# Implementation of a Prospective Index-Cluster Sampling Strategy for the Detection of Presymptomatic Viral Respiratory Infection in Undergraduate Students

**DOI:** 10.1093/ofid/ofae081

**Published:** 2024-02-14

**Authors:** Diya M Uthappa, Micah T McClain, Bradly P Nicholson, Lawrence P Park, Ilya Zhbannikov, Sunil Suchindran, Monica Jimenez, Florica J Constantine, Marshall Nichols, Daphne C Jones, Lori L Hudson, L Brett Jaggers, Timothy Veldman, Thomas W Burke, Ephraim L Tsalik, Geoffrey S Ginsburg, Christopher W Woods

**Affiliations:** Doctor of Medicine Program, Duke University School of Medicine, Durham, North Carolina, USA; Duke Global Health Institute, Duke University, Durham, North Carolina, USA; Center for Infectious Disease Diagnostics and Innovation, Duke University Medical Center, Durham, North Carolina, USA; Durham Veterans Affairs Health Care System, Durham, North Carolina, USA; Institute for Medical Research, Durham, North Carolina, USA; Duke Global Health Institute, Duke University, Durham, North Carolina, USA; Center for Infectious Disease Diagnostics and Innovation, Duke University Medical Center, Durham, North Carolina, USA; Durham Veterans Affairs Health Care System, Durham, North Carolina, USA; Bioinformatics and Clinical Analytics Team, Clinical Research Unit, Duke University Department of Medicine, Durham, North Carolina, USA; Center for Infectious Disease Diagnostics and Innovation, Duke University Medical Center, Durham, North Carolina, USA; Institute for Medical Research, Durham, North Carolina, USA; Center for Infectious Disease Diagnostics and Innovation, Duke University Medical Center, Durham, North Carolina, USA; Duke Institute for Health Innovation, Durham, North Carolina, USA; Durham Veterans Affairs Health Care System, Durham, North Carolina, USA; Duke Clinical Research Institute, Duke University School of Medicine, Durham, North Carolina, USA; Center for Infectious Disease Diagnostics and Innovation, Duke University Medical Center, Durham, North Carolina, USA; Duke Global Health Institute, Duke University, Durham, North Carolina, USA; Center for Infectious Disease Diagnostics and Innovation, Duke University Medical Center, Durham, North Carolina, USA; Center for Infectious Disease Diagnostics and Innovation, Duke University Medical Center, Durham, North Carolina, USA; Durham Veterans Affairs Health Care System, Durham, North Carolina, USA; Center for Infectious Disease Diagnostics and Innovation, Duke University Medical Center, Durham, North Carolina, USA; Duke Global Health Institute, Duke University, Durham, North Carolina, USA; Center for Infectious Disease Diagnostics and Innovation, Duke University Medical Center, Durham, North Carolina, USA; Durham Veterans Affairs Health Care System, Durham, North Carolina, USA

**Keywords:** college health, disease transmission, respiratory viral illness, surveillance

## Abstract

**Background:**

Index-cluster studies may help characterize the spread of communicable infections in the presymptomatic state. We describe a prospective index-cluster sampling strategy (ICSS) to detect presymptomatic respiratory viral illness and its implementation in a college population.

**Methods:**

We enrolled an annual cohort of first-year undergraduates who completed daily electronic symptom diaries to identify index cases (ICs) with respiratory illness. Investigators then selected 5–10 potentially exposed, asymptomatic close contacts (CCs) who were geographically co-located to follow for infections. Symptoms and nasopharyngeal samples were collected for 5 days. Logistic regression model–based predictions for proportions of self-reported illness were compared graphically for the whole cohort sampling group and the CC group.

**Results:**

We enrolled 1379 participants between 2009 and 2015, including 288 ICs and 882 CCs. The median number of CCs per IC was 6 (interquartile range, 3–8). Among the 882 CCs, 111 (13%) developed acute respiratory illnesses. Viral etiology testing in 246 ICs (85%) and 719 CCs (82%) identified a pathogen in 57% of ICs and 15% of CCs. Among those with detectable virus, rhinovirus was the most common (IC: 18%; CC: 6%) followed by coxsackievirus/echovirus (IC: 11%; CC: 4%). Among 106 CCs with a detected virus, only 18% had the same virus as their associated IC. Graphically, CCs did not have a higher frequency of self-reported illness relative to the whole cohort sampling group.

**Conclusions:**

Establishing clusters by geographic proximity did not enrich for cases of viral transmission, suggesting that ICSS may be a less effective strategy to detect spread of respiratory infection.

## Teaser

In a college population, an index-cluster sampling strategy in which clusters were established based on geographic proximity alone did not enrich for cases of respiratory viral transmission.

Acute respiratory illnesses (ARIs) are a frequent cause of morbidity in college populations, with >90% of students experiencing at least 1 ARI in an academic year [1]. Despite the apparent frequency of illness, epidemiologic data on ARI among college populations are lacking, with the notable exceptions of the severe acute respiratory syndrome coronavirus 2 (SARS-CoV-2) pandemic [2, 3] and influenza [4–6].

College campuses are important sources for community spread of infection, both within the university setting but also to the surrounding community as students go off campus or return home for breaks [7]. Understanding the spread of ARI in this population can inform public health policies on campus and highlight potential risks to the local community [8]. The SARS-CoV-2 pandemic has highlighted the need to expediently identify and monitor exposed individuals who may shed virus before they develop symptoms. However, little is known about transmission networks on campus, particularly as they relate to this early time window.

For those who live in congregate settings, early and accurate identification of at-risk persons enables early treatment as well as isolation/quarantine strategies. The postexposure, presymptomatic period (ie, latent period) is critically important to achieve these goals but also hardest to identify given that people are by definition asymptomatic. Whereas patient-reported symptoms may not identify this latent phase, host response measurements (eg, gene expression changes) are highly accurate during this window [9–11]. In prior work, we discovered a host gene expression signature that identified presymptomatic viral infection in a human challenge experimental model [9, 10]. We subsequently validated that model in an index-cluster design enrolling Duke University undergraduates over several years [12]. In the present study, we present the methodology associated with that prospective index-cluster sampling strategy (ICSS). Specifically, we describe the ability of an ICSS study to identify respiratory viral transmission during the presymptomatic phase.

## METHODS

### Setting and Participants

We performed syndromic and diagnostic surveillance for viral ARI in a population of young adults (aged ≥18 years) living in first-year group housing on campus at Duke University in Durham, North Carolina. First-year housing consists of primarily double-occupancy rooms with shared bathrooms and a common living area. At the beginning of each academic year from 2009 to 2015, investigators approached first-year students through voluntary information sessions. For the 2013–2014 year, enrollment only occurred during the spring semester (January). Informed consent was obtained from interested students. No enrollment took place during the 2012–2013 academic year due to funding limitations.

### Patient Consent Statement

The study was approved by the Duke University Office of Student Affairs and the Duke University Medical Center Institutional Review Board (Pro00001176) and was conducted in accordance with the Declaration of Helsinki.

### Data and Sample Collection

#### Enrollment

At the enrollment visit, participants completed a standardized questionnaire (Supplementary Appendix A) including demographic information (age, gender, race/ethnicity, dormitory location) and information about behavioral risk factors, including smoking history and influenza immunization.

#### Symptom Monitoring

Assessment of self-reported illness for each participant in the cohort was determined from the daily symptom diary (Supplementary Appendix B). A web-based instrument (administered via Blackboard [Anthology Inc, Boca Raton, Florida] from 2009 to 2012 and via Qualtrics [Qualtrics XM, Seattle, Washington] from 2013 to 2015) was used by the participants to record daily assessments of respiratory illness based on 8 symptoms: nasal discharge, nasal congestion, sneezing, malaise, cough, sore throat, fever/chills, and headache. Each symptom was graded on a severity scale from 0–4, with 0 indicating not present, 1 indicating mild symptoms, 2 indicating moderate symptoms, 3 indicating severe symptoms, and 4 indicating very severe symptoms [12]. From these scores, a cumulative modified Jackson score was calculated as the sum of all 8 symptoms, with value ranging from 0 to 32 [13].

#### Index Case Definition and Identification

The completed symptom surveys were monitored daily by research personnel. A participant was identified as a potential index case (IC) when they reported an increase in the cumulative daily symptom monitoring score of >6 compared to the median score in the preceding 7-day window [12]. For example, a participant with a median score of 0 in the preceding week would need a score of 6 to qualify. A participant with a median score of 5 in the preceding week would need a score of 11 to qualify as a potential IC. Upon confirmation of these symptoms by research staff, nasopharyngeal (NP) and blood samples were collected for testing (see “Biological Sample Collection” below).

#### Close Contact Case Definition and Identification

Upon symptom development in 1 or more ICs in a living area, investigators identified a cluster (typically 5–10 participants) of asymptomatic close contacts (CCs), defined as previously enrolled, asymptomatic participants living within the same dormitory (eg, roommates, neighbors, same floor, same building as the IC). A participant could be identified as a CC for multiple ICs if the ICs in question were symptomatic within 1 day of each other. Following identification, the symptom scores of CCs were monitored for up to 5 consecutive days for development of illness. Once again, cumulative daily symptom score of >6 compared to the median score in a previous 7-day window was used as the threshold for subjective symptomatic illness. Participants being monitored as CCs could later become ICs only after the 5-day monitoring period elapsed. CC biological sample collection involved completion of an enhanced data collection form for exposures and symptoms as well as collection of NP swabs and whole blood samples daily for up to 5 consecutive days.

#### Biological Sample Collection

Blood samples were collected as previously described [12] but were not used for this study. NP swab samples were polymerase chain reaction (PCR) tested for the presence of respiratory viral pathogens using either the ResPlex II Panel v2.0 (Qiagen, Hilden, Germany) or xTAG (Luminex, Austin, Texas). The panels assessed for influenza A and B, adenovirus, bocavirus, parainfluenza 1–4, respiratory syncytial virus A and B, human metapneumovirus, human coronavirus (hCoV; HKU1, NL63, 229E, OC43), coxsackievirus/echovirus, and rhinovirus. For convenience, the subset of CCs selected for biological assessment were primarily those associated with an IC who had developed symptomatic illness on a Friday, Saturday, or Sunday. This facilitated the collection of CC data as the necessary biological sample collection could be completed over the following work week (Monday–Friday).

#### Closeout Visit

At a closeout visit conducted at the end of each school year, participants completed a questionnaire (Supplementary Appendix C) to provide information describing their exposures and symptom assessment of the preceding academic year including information on influenza immunizations and how many episodes of illness the student recalled.

### Statistical Analysis

Descriptive statistics were calculated separately for each of the 4 academic years as well as for the cohort overall.

We investigated the ability of an ICSS to enrich for early identification of secondary symptomatic illness. To do this, we compared the proportion of participants who reported symptomatic illness in the CC group to that of the whole cohort sampling group for each week over the course of each academic year. The whole cohort sampling group included all participants who completed symptom monitoring during the week of interest, while those in the CC sampling group were only those participants identified as CC by the start of the week. Given that a participant's CC status varied on a week-by-week basis and depended on whether they were in geographic proximity to an IC, the participants in the CC sampling group varied weekly. To compare symptomatic illness between the whole cohort and the CC group, the proportion of symptomatic illness occurring in each group was calculated by academic year using a logistic regression model. Absence of a symptom report was not imputed as an absence of symptoms.

To assess the impact of imputing a symptom score of 0 where there was an absence of symptom reports, we conducted a sensitivity analysis to compare attack rates across each week among the following groups: the CC group, those in the whole cohort who completed at least 25% of symptoms logs without absent symptom log imputation, and those in the whole cohort who completed at least 25% of symptoms logs where the lack of a symptom report was imputed as a symptom score of 0. A symptom report completion rate of 25% was selected as a cutoff to maximize both the number of participants included and the completeness of their longitudinal record.

## RESULTS

### Demographic Data

A total of 1379 participants were enrolled in the surveillance cohort, with the number of students enrolled ranging from 105 to 448 per academic year. Students had a median age of 18 (range, 18–25) years; 60% were White, 28% Asian, 9.6% Black/African American, and 9% Hispanic, and 50.6% were female (Table 1).

**Table 1.** Demographics and Respiratory Symptoms of a College First-year Cohort, 2009–2012 and 2014–2015, North Carolina

Characteristic	Total (N = 1379)	Academic Year			
		2009–2010 (n = 448)	2010–2011 (n = 406)	2011–2012 (n = 420)	2014–2015 (n = 105)^a^
Age, y, mean (range)	18.2 (18–25)	18.3 (18–22)	18.2 (18–25)	18.2 (18–21)	18.3 (18–25)
Female gender, No. (%)	698 (50.6)	218 (48.7)	188 (46.3)	238 (56.7)	54 (51.4)
Race/Ethnicity, No. (%)^b^					
White	828 (60.0)	266 (59.4)	237 (58.4)	258 (61.4)	67 (63.8)
Asian	386 (28.0)	115 (25.7)	114 (28.1)	132 (31.4)	25 (23.8)
African American	133 (9.6)	38 (8.5)	42 (10.3)	37 (8.8)	16 (15.2)
Hispanic	123 (9.0)	39 (8.7)	34 (8.4)	38 (9.4)	12 (11.5)
Current or occasional smoker, No. (%)	61 (6.4)	18 (4.1)	440 (9.9)	NC	3 (2.9)
Flu vaccine up to date, No. (%)	189 (20.3)	81 (18.2)	75 (18.8)	NC	33 (36.7)
Total No. of surveys/symptom days	95 966	29 495	27 032	29 841	9581
Symptom days reported, median (IQR)	70 (25–118)	64 (23–115)	69 (21–115)	69 (28–118)	104 (38–140)
Total daily symptom score, median (IQR)	1 (0–4)	0 (0–3)	1 (0–4)	1 (0–4)	1 (0–5)

Abbreviations: IQR, interquartile range; NC, not counted.

^a^Financial constraints limited participant enrollment during this academic year.

^b^Participants were able to select multiple options when identifying race/ethnicity.

### Epidemiological Assessment of ARI

#### Surveillance for ARI Events Overall

Of the 1379 enrolled participants, 1301 (94%) completed at least 1 daily symptom survey. For the duration of the study period, 95 949 daily symptom surveys were submitted with a median of 70 (interquartile range [IQR], 25–118) symptom surveys per individual. The median reported symptom score was 1 (IQR, 0–4). Table 1 presents details by academic cohort. Comparing the distribution of symptom scores across the 2009–2010, 2010–2011, and 2011–2012 cohorts, the greatest proportion of reported symptom-free days (days with a symptom score of 0) occurred during the 2009–2010 academic year, which was the academic year coinciding with the H1N1 pandemic (Supplementary Table 1).

On the closeout visit surveys conducted at the end of each academic year, 72.3% of participants recalled having at least 1 ARI, 50% recalled at least 2, 21.4% recalled ≥3, and 9.7% recalled ≥4 episodes (Supplementary Table 2). For the 2009–2010 cohort, we compared participants’ recall of the number of episodes of ARI experienced to the number of episodes logged via symptom diaries and found that in 27% of participants (59/217), the number recalled matched the number logged. In 63% of cases (138/217), the number recalled was within 1 episode of those logged (Supplementary Figure 1).

#### Index Case ARI Analysis

Of the 1379 participants, investigators selected 288 potential ICs for evaluation of incident ARI. Blood and NP samples were collected for 246 (85%) of these individuals. A putative viral etiology was identified in the majority of cases (57%). The annual pattern of illness among ICs (self-reported symptomatic events and PCR-confirmed viral events) is shown in Supplementary Figure 2. The most commonly identified virus was rhinovirus (18% of all those IC tested; 31% of positive IC) (Table 2). Supplementary Figure 3 is a presentation of the seasonality of PCR-confirmed viral events among ICs by academic year and virus type, and Supplementary Figure 4 summarizes the seasonality of PCR-confirmed viral events for the most commonly identified viruses. Both figures highlight an increased frequency of viral events occurring in the fall and winter, coinciding with the return of students to campus from prolonged breaks.

**Table 2.** Respiratory Virus Etiologies Among Index Cases in a College First-year Cohort, 2009–2012 and 2014–2015, North Carolina

Etiology	All Years (N = 246)	2009–2010 (n = 81)	2010–2011 (n = 87)	2011–2012 (n = 47)	2014–2015 (n = 31)
No analyte detected	106 (43.1)	33 (40.7)	41 (47.1)	17 (36.2)	15 (48.4)
Adenovirus	3 (1.2)	0 (0.0)	1 (1.2)	0 (0.0)	2 (6.5)
Bocavirus	1 (0.4)	0 (0.0)	0 (0.0)	0 (0.0)	1 (3.2)
Coronavirus	27 (11.0)	3 (3.7)	11 (12.6)	6 (12.8)	7 (22.6)
Coxsackievirus/echovirus	26 (10.6)	11 (13.6)	11 (12.6)	4 (8.5)	0 (0.0)
Human metapneumovirus	8 (3.3)	2 (2.5)	0 (0.0)	3 (6.4)	3 (9.7)
Influenza A	17 (6.9)	10 (12.4)	7 (8.0)	0 (0.0)	0 (0.0)
Parainfluenza	7 (2.8)	3 (3.7)	0 (0.0)	3 (6.4)	1 (3.2)
RSV	7 (2.8)	1 (1.2)	3 (3.4)	3 (6.4)	0 (0.0)
Rhinovirus	44 (17.9)	18 (22.2)	13 (14.9)	11 (23.4)	2 (6.5)
Coinfections	13 (5.3)	5 (6.1)	7 (8.0)	1 (2.1)	0 (0.0)

Data are presented as No. (%).

Abbreviation: RSV, respiratory syncytial virus.

The 143 ICs with a positive viral PCR demonstrated a higher median symptom score (15 [IQR, 11–20]) than the 106 ICs without a virus detected (13 [IQR, 11–18]) (Wilcoxon rank-sum *P* = .022). Median symptom scores were highest for ICs with influenza A, with a score of 21 (IQR, 14–25), and were lowest for participants with hCoV or coxsackievirus/echovirus (13 [IQR, 8–19] and 13.5 [IQR, 11–19]), respectively (Supplementary Table 3).

#### Close Contact ARI Analysis

The 249 ICs generated 882 CCs. The median number of close contacts per cluster was 6 (IQR, 3–8). During the CCs’ 5-day monitoring period, the median number of days for symptom survey completion was 4 (IQR, 4–5). The symptoms in the CCs were mild with a mean peak cumulative symptom score of 2.8 (median, 1 [IQR, 0–4]). Among the 882 CCs, 111 (13%) of the participants developed self-reported symptomatic ARI during the monitoring period.

Biological sample collection and viral testing were obtained for 60 of the IC-CC clusters, which included 60 ICs and 719 CCs overall. During the 5-day monitoring period, the median number of days for biospecimen collection was 4 (IQR, 4–5). Virus was detected in 37 (62%) of the ICs and 106 of the CCs (15%). Regarding viral concordance between the ICs and their associated CCs, there were 19 CCs (18% of positive CC) who tested positive for the same virus as their associated IC: 10 with rhinovirus, 7 with coxsackievirus/echovirus, and 2 with hCoV. In the other 87 CC cases with test-positive viral ARI, the specific pathogen was different from that found in the linked IC.

Of the CCs tested, 85% had no detectable virus. Of the CCs that tested positive, 43% (46/106) were positive for rhinovirus, 25% (27/106) for coxsackievirus/echovirus, and 13% (14/106) for hCoV (Table 3). Eight coinfections were identified and represented combinations of coxsackievirus/echovirus, rhinovirus, hCoV, or human metapneumovirus.

**Table 3.** Respiratory Virus Etiologies Among Close Contacts in a College First-year Cohort, 2009–2012 and 2014–2015, North Carolina

Characteristic	All Years (N = 719)	2009–2010 (n = 155)	2010–2011 (n = 159)	2011–2012 (n = 180)	2014–2015 (n = 225)
No analyte detected	613 (85.3)	125 (80.6)	148 (93.1)	149 (82.8)	191 (84.9)
Adenovirus	5 (0.7)	0 (0.0)	0 (0.0)	0 (0.0)	5 (2.2)
Bocavirus	2 (0.3)	0 (0.0)	0 (0.0)	0 (0.0)	2 (0.9)
Coronavirus	14 (2.0)	7 (4.5)	2 (1.3)	1 (0.6)	4 (1.8)
Coxsackievirus/echovirus	27 (3.8)	11 (7.1)	6 (3.8)	10 (5.6)	0 (0.0)
Human metapneumovirus	3 (0.4)	1 (0.6)	0 (0.0)	0 (0.0)	2 (0.9)
Influenza	3 (0.4)	1 (0.7)	0 (0.0)	1 (0.6)	1 (0.4)
Parainfluenza	2 (0.3)	0 (0.0)	1 (0.6)	1 (0.6)	0 (0.0)
RSV	4 (0.6)	1 (0.6)	0 (0.0)	0 (0.0)	3 (1.3)
Rhinovirus	46 (6.4)	9 (5.8)	2 (1.3)	18 (10.0)	17 (7.6)
Coinfection	6 (0.8)	3 (1.9)	2 (1.3)	0 (0.0)	1 (0.4)

Data are presented as No. (%).

Abbreviation: RSV, respiratory syncytial virus.

The median peak symptom score for the CCs with associated etiology testing was 1 (IQR, 0–4). The median peak symptom scores associated with positive and negative viral detection were 5 (IQR, 1–9) and 1 (IQR, 0–3), respectively. Overall, among those CCs with positive viral detection, symptom severity was highest in the 3 CCs in which human metapneumovirus was detected (median, 8 [IQR, 2–9]) and lowest in the 21 CCs positive for coxsackievirus/echovirus (median, 2 [IQR, 0–7]).

### Evaluation of the ICSS

**Figure 1.**
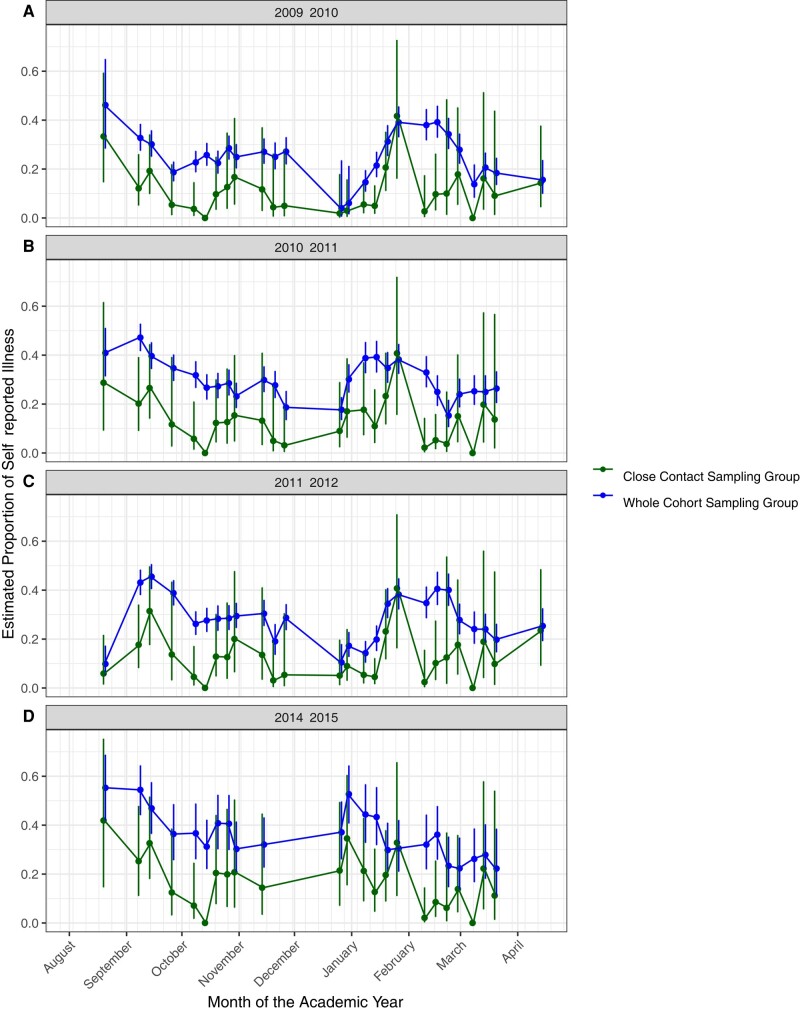
Comparison of symptomatic illness between the whole cohort and the close contact (CC) group using logistic regression analysis. The CC sampling group is represented in green, and whole cohort sampling group is represented in blue. Weekly point estimates for the proportion of self-reported illness have been graphed along with the corresponding 95% confidence intervals. The sparse reporting midyear corresponds to winter break.

**Figure 2.**
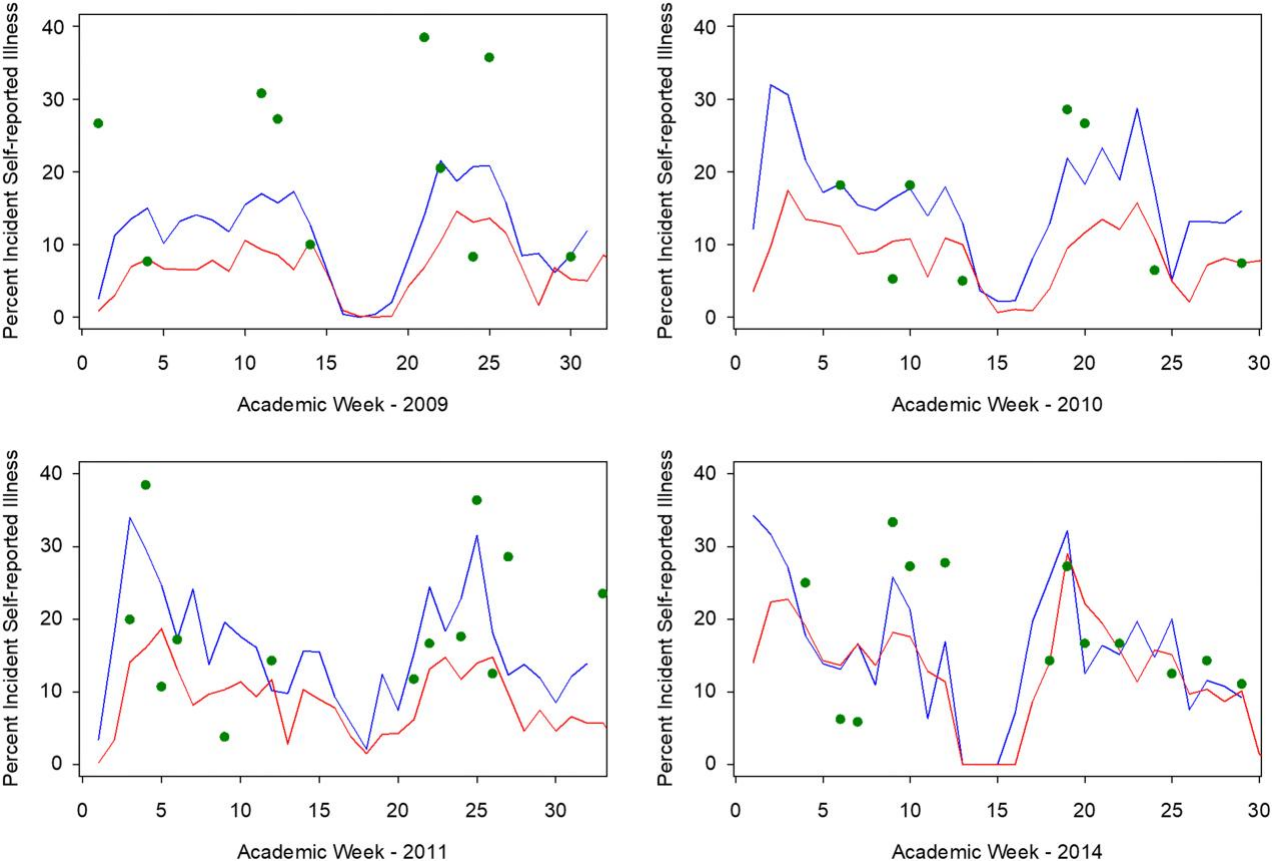
Sensitivity analysis: comparison of symptomatic illness between the whole cohort and the close contact (CC) group, with and without imputation of missing symptom reports as an absence of symptoms. Green dots represent the attack rate among the CCs. Blue line represents the attack rate among those in the whole cohort who completed at least 25% of symptom logs without imputation. Red line represents the attack rate using the imputed sensitivity analysis data set—the attack rate among those in the whole cohort who completed at least 25% of symptom logs where the lack of a symptom report was imputed as a symptom score of 0.

There were 399 cases of symptomatic illnesses reported: 288 among those who self-identified as ICs and 111 in the 882 CCs during their 5-day monitoring period. Week-by-week comparison of the proportion of symptomatic illness occurring in the CC group compared to the proportion of symptomatic illness occurring in the whole participant cohort showed that for the majority of weeks in each academic year, the proportion of symptomatic illness identified was no different within the CC sampling group compared to the whole participant cohort (Figure 1). If the ICSS enriched for respiratory viral transmission events, we hypothesized that the rate of ARI among CCs would be higher than the background rate of ARI observed in the general study population. However, the rates were similar for the CCs compared to the whole cohort across each academic year. Furthermore, the sensitivity analysis comparing attack rates between CCs and the whole cohort with and without imputation of missing symptom reports as an absence of symptoms showed no difference in attack rate between the CC group and either whole cohort group (Figure 2). Overall, ICSS did not facilitate increased identification of respiratory viral transmission.

## DISCUSSION

In a prospective ICSS, we have characterized the etiological distribution, symptom severity, and frequency of viral ARI within a cohort of generally healthy young adults in congregate living spaces across 4 academic years prior to the coronavirus disease 2019 (COVID-19) pandemic. Our study's aim of collecting respiratory viral illness data from young adults in a community-type college setting adds to the body of ARI research in which secondary sampling of patient populations who are inpatient or have presented to the emergency department for care is overrepresented [14]. Moreover, presentation of data from the 2009–2010 academic year, which coincided with the HIN1 global pandemic, and the subsequent academic years provides insight into how college respiratory viral epidemiology shifts during and after a global pandemic. For example, for the 2009–2010 participant cohort, the number of symptom-free days (symptom score of 0 recorded by participants) was greater than in both the postpandemic 2010–2011 and 2011–2012 student cohorts. This trend resembles the reduced impact of respiratory viral diseases (most notably influenza) driven by social behaviors (increased social distancing, hand hygiene, masking) during the winter 2020–2021 of the COVID-19 pandemic [15]. However, our finding from 2009–2010 is likely due to H1N1 circulation peaking prior to the bulk of the academic year [16, 17], rather than the widely enacted infection prevention behaviors, which played a role in reduced respiratory viral illness circulation during the winter of 2020–2021 [15].

Additionally, we found that within a college population, an ICSS that links CCs to ICs by geographic proximity alone does not meaningfully enrich for identification of cases of presymptomatic illness when compared to whole cohort sampling. This finding may be attributable to late identification of IC, which may in turn preclude meaningful selection of CCs. Additionally, it may also be a result of the diversity and frequency of interactions outside of the CC group in a college population (classes, social events, affinity groups, athletic teams, etc). This is further highlighted by the lack of viral concordance between ICs and CCs with positive viral detection. Concurrent viral transmission and sporadic endemic virus circulation could account for differences in detected virus between ICs and CCs on a college campus and may limit use of an ICSS that geographically links ICs and CCs. However, in situations where living groups and social interactions are more fixed, such as in regions with strictly enforced COVID time lockdowns or household studies of ARI [18, 19], an ICSS with geographic linkages between ICs and CCs may have increased utility and yield results more closely resembling those hypothesized. Overall, on a college campus where respiratory viral burden is high, endemic virus circulation is sporadic, and interpersonal interactions are numerous, random selection of individuals rather than a geographically mediated ICSS may be a simpler way to identify cases of presymptomatic illness.

Continued efforts must be made toward identifying novel surveillance strategies for the early detection of presymptomatic respiratory viral disease. Doing so is important for reducing disease transmission and essential for early intervention. As evidenced by the current COVID-19 pandemic, exploring surveillance methods that increase early detection and reduce spread of viral respiratory pathogens is especially important among the college population living in congregate settings. Over the summer of 2020 alone, people aged 20–29 accounted for >20% of all confirmed COVID-19 cases, representing the age bracket with the highest incidence [20]. In October 2020, the Centers for Disease Control and Prevention highlighted younger adults as likely contributors to community transmission of COVID-19. Given that a large proportion of college students fall within this age range, innovative study designs that facilitate efficient identification of postexposure, presymptomatic, and asymptomatic secondary cases and epidemiological investigations of viral ARI on college campuses are important to maintain college functioning and similar congregate living environments such as skilled nursing facilities and group homes, as well as limiting transmission of respiratory viral pathogens to the community at large.

Our study had various strengths. It was conducted prospectively over 5 years, permitting robust collection of descriptive data within a cohort of college first-years. Despite ARI being a major cause of morbidity within the college population [1], the breadth of respiratory viral illness epidemiology within this sizable population [21] of degree-seeking students is lacking. Through a combination of both self-reported data and etiological testing, this study enables a closer look at respiratory viral illness within this underrepresented population with regard to ARI. However, our study has several limitations. First, over the extended time period in which the study took place, we did have gaps in symptom reporting and in the response rate to close out surveys. Failure to complete daily symptom monitoring logs may have resulted in participants underreporting asymptomatic days and as a result, artificially inflating the proportion of participants logging symptoms qualifying as self-reported illness. Second, our determination of a respiratory illness was based on self-reported symptom logs and will not capture low or asymptomatic infections. In relying on self-entered symptom logs over objective measures for viral detection, there is the potential for misclassification of illness. As a result, if those participants more likely to complete daily symptom monitoring are also those who are more likely to notice and log smaller relative changes in daily symptoms, the response data collected could be biased toward higher rates of illness among the participant cohort. Last, we were not able to tie IC-CC clusters by time; if exposure between index case and a close contact occurred, the time of exposure was not known. Despite these limitations, we successfully collected a large volume of reliable data using a novel study design to gain valuable insight in college populations.

## CONCLUSIONS

College viral respiratory illness data collected longitudinally and prior to the onset of the COVID-19 pandemic can provide a baseline for detecting lasting changes in college respiratory viral illness epidemiology following this pandemic. Furthermore, given the sporadic circulation of endemic respiratory viruses within a college-dormitory setting, an ICSS that links CCs to ICs by geographic proximity alone does not meaningfully enrich for disease transmission events.

## Supplementary Material

ofae081_Supplementary_Data

## Supplementary Data

Supplementary materials are available at *Open Forum Infectious Diseases* online. Consisting of data provided by the authors to benefit the reader, the posted materials are not copyedited and are the sole responsibility of the authors, so questions or comments should be addressed to the corresponding author.

## Notes

***Acknowledgments.*** We thank Debra Freeman, Bartlett Humphries, Sara Hoffman, Anna Mazur, Olga M. Better, Mert Aydin, Pamela Isner, Stephanie Dobos, Samia Baluch, Andrew Burd, Kyle R. Breitschwert, and Ashlee M. Valente for enrolling participants, processing samples and data, and engaging with the undergraduate campus. Furthermore, we would like to thank the students of Duke University.

***Disclaimer.*** The statements and conclusions herein are those of the authors and do not reflect the position or policy of the US government.

***Financial support***. This study was funded by the US Defense Advanced Research Projects Agency (projects N66001-07-C-2024 and W911NF1410052). D. M. U. received funding from the Eugene A. Stead Scholarship, Duke University School of Medicine.
