# Supplementary material for: Implementation of a Prospective Index-Cluster Sampling Strategy for the Detection of Presymptomatic Viral Respiratory Infection in Undergraduate Students
Source: Open Forum Infect Dis. 2024 Feb 14;11(3):ofae081. doi: 10.1093/ofid/ofae081 (PMC10911223; doi:10.1093/ofid/ofae081)
Supplement: ofae081_Supplementary_Data [file ofae081_supplementary_data.zip › Student Study_OFID_supp material_240121.docx]

**SUPPLEMENTARY MATERIALS**

**SUPPLEMENTAL TABLES**

- **Supplemental Table 1: A comparison of symptom days by severity across several of the yearly cohorts**
- **Supplemental Table 2: Participant Recall of Respiratory Illness on Closeout Survey**
- **Supplemental Table 3: Evaluation of Index Case symptom scores (Modified Jackson Score) by respiratory viral etiology.**

**SUPPLEMENTAL FIGURES**

- **Supplemental Figure 1. Comparison of Reported vs Recalled Episodes of Illness Among Students from the 2009-2010 cohort**
- **Supplemental Figure 2. Annual pattern of illness among Index Cases by academic year**
- **Supplemental Figure 3. Seasonality of PCR-confirmed viral events among Index Cases by academic year and virus-type**
- **Supplemental Figure 4. Seasonality of PCR-confirmed viral events among Index Cases for the three most commonly identified viruses**

**APPENDICES**

- **Appendix A. Enrollment Questionnaire**
- **Appendix B. Daily Symptom Diary**
- **Appendix C. Closeout Questionnaire**

**SUPPLEMENTAL TABLES:**

**Supplemental Table 1: A comparison of symptom days by severity across several of the yearly cohorts**

|  | Severe Symptom- Days  (Daily symptom score of 20+)  N (%) | Moderate Symptom- Days  (Daily symptom score of 13-19)  N (%) | Mild Symptom- Days  (Daily symptom score of 7-12)  N (%) | Minor Symptom-Days  (Daily symptom score of 1-6)  N (%) | Day Recorded without Symptoms  (Daily symptom score of 0)  N (%) | Total Symptom- Days Recorded |
| --- | --- | --- | --- | --- | --- | --- |
| 2009 – 2010 | 255  (0.8%) | 838  (2.8%) | 2519  (8.3%) | 11202  (37.0%) | 15423 (51.0%) | 30237 |
| 2010 – 2011 | 299  (1.1%) | 1032  (3.7%) | 2918  (10.4%) | 10828  (38.8%) | 12852  (46.0%) | 27929 |
| 2011 – 2012 | 481  (1.6%) | 1312  (4.3%) | 3234  (10.6%) | 11487  (37.6%) | 13996  (45.9%) | 30510 |

**Supplemental Table 2: Participant Recall of Respiratory Illness on Closeout Survey**

|  | 2009- 2010 | 2010-2011 | 2011-2012 | SPRING 2014 | 2014-2015 | ALL YEARS |
| --- | --- | --- | --- | --- | --- | --- |
| Number of enrolled subjects who completed closeout surveys | 253  (%) | 226  (%) | 246  (%) | 69  (%) | 72  (%) | 866  (%) |
| Number of Episodes of Respiratory Sickness Recalled over the Academic Year |  |  |  |  |  |  |
| 0 | 6  (2.4) | 6  (2.7) | 96  (39.0) | 4  (5.8) | 5  (6.9) | 117  (13.5) |
| 1 | 53  (21.0) | 43  (19.0) | 64  (26.0) | 19  (27.5) | 14  (19.4) | 193  (22.3) |
| 2 | 74  (29.3) | 71  (31.4) | 59  (24.0) | 19  (27.5) | 25  (34.7) | 248  (28.6) |
| 3 | 29  (11.5) | 24  (10.6) | 19  (7.7) | 13  (18.8) | 16  (22.2) | 101  (11.7) |
| 4 | 20  (7.9) | 22  (9.7) | 8  (3.3) | 0  (0) | 0  (0) | 50  (5.8) |
| >4 | 13  (5.14) | 7  (3.1) | 0  (0.0) | 4  (5.8) | 10  (13.9) | 34  (3.9) |
| Cannot Recall | 58  (22.9) | 53  (23.5) | 0  (0) | 10  (14.5) | 2  (2.8) | 123  (14.2) |

**Supplemental Table 3: Evaluation of Index Case symptom scores (Modified Jackson Score) by respiratory viral etiology.**

|  | Number of ICs | Total Symptom Score  Mean (SD) | Rhinorrhea  (SD) | Congestion  (SD) | Sneezing  (SD) | Cough  (SD) | Malaise  (SD) | Sore Throat  (SD) | Fever  (SD) | Headache  (SD) |
| --- | --- | --- | --- | --- | --- | --- | --- | --- | --- | --- |
| All Index Cases | 249 | 15.2  (5.6) | 2.1  (1.1) | 2.2  (1.0) | 1.4  (1.0) | 2.3  (1.2) | 2.4  (1.0) | 2.3  (1.1) | 1.0  (1.1) | 1.5(1.2) |
| No Analyte Detected | 106 | 15.0  (5.5) | 1.8  (1.0) | 2.1  (1.0) | 1.2  (1.0) | 2.2  (1.2) | 2.3  (1.0) | 2.4  (1.1) | 1.2  (1.2) | 1.7  (1.2) |
| Analyte Detected | 143 | 16.1  (5.8) | 2.4  (1.0) | 2.4  (1.0) | 1.7  (1.0) | 2.4  (1.1) | 2.5  (1.1) | 2.2  (1.1) | 0.9  (1.1) | 1.4  (1.2) |
| Adenovirus | 3 | 12.8  (8.2) | 2.0  (0.8) | 1.8  (0.5) | 0.5  (0.6) | 2.3  (1.5) | 2.0  (1.6) | 1.8  (1.5) | 1.5  (1.7) | 1.0  (1.4) |
| Bocavirus^a^ | 1 | 8.0 | 2.0 | 2.0 | 1.0 | 1.0 | 1.0 | 1.0 | 0.0 | 0.0 |
| Coronavirus | 27 | 14.7  (6.3) | 2.5  (0.9) | 2.4  (0.8) | 1.6  (1.0) | 1.9  (1.3) | 2.2  (1.1) | 1.9  (1.2) | 0.9  (1.1) | 1.2  (1.3) |
| Coxsackie/  echovirus | 26 | 15.7  (5.8) | 2.5  (0.9) | 2.7  (1.1) | 1.7  (1.0) | 2.4  (1.2) | 2.5  (1.2) | 2.0  (1.1) | 0.6  (0.8) | 1.2  (1.1) |
| Human Metapneumovirus | 8 | 18.4  (3.3) | 2.8  (0.9) | 2.3  (0.9) | 2.3  (0.9) | 2.7  (1.1) | 3.2  (0.8) | 2.3  (0.8) | 1.0  (0.9) | 1.8  (1.0) |
| Influenza A | 17 | 20.8  (7.3) | 2.3  (1.1) | 2.7  (1.0) | 1.9  (1.1) | 3.4  (0.7) | 2.9  (1.2) | 3.0  (1.0) | 2.1  (1.5) | 2.4  (1.7) |
| Parainfluenza | 7 | 13.3  (3.4) | 1.7  (1.2) | 1.7  (0.5) | 1.8  (1.0) | 2.2  (0.4) | 2.2  (0.4) | 2.3  (0.8) | 0.7  (0.8) | 0.8  (0.8) |
| RSV | 7 | 16.8  (4.5) | 2.7  (1.2) | 2.7  (0.8) | 1.2  (1.3) | 2.3  (1.6) | 3.2  (0.4) | 3.0  (0.9) | 0.5  (0.8) | 1.3  (1.2) |
| Rhinovirus | 44 | 15.9  (5.1) | 2.4  (1.0) | 2.4  (1.0) | 1.8  (1.0) | 2.5  (0.9) | 2.4  (0.9) | 2.2  (1.0) | 0.7  (0.8) | 1.4  (1.1) |
| Coinfection | 13 | 16.2  (5.0) | 2.7  (0.9) | 2.6  (0.9) | 2.1  (0.9) | 2.6  (1.0) | 2.5  (1.0) | 2.0  (0.9) | 0.6  (0.8) | 1.1  (1.0) |

^a^ Given N of 1, unable to calculate standard deviation.

**SUPPLEMENTAL FIGURES**

**Supplemental Figure 1. Comparison of Reported vs Recalled Episodes of Illness Among Students from the 2009-2010 cohort**

Negative numbers indicate people who recalled more episodes of illness in their year-end survey than they actually reported over the course of the year.

**Supplemental Figure 2. Annual pattern of illness among Index Cases by academic year**

The purple line corresponds to the number of cases of self-reported illness among ICs. The green points correspond to the number of cases of viral illnesses detected by PCR-testing among ICs.

**Supplemental Figure 3. Seasonality of PCR-confirmed viral events among Index Cases by academic year and virus-type**

**Supplemental Figure 4. Seasonality of PCR-confirmed viral events among Index Cases for the three most commonly identified viruses**

**
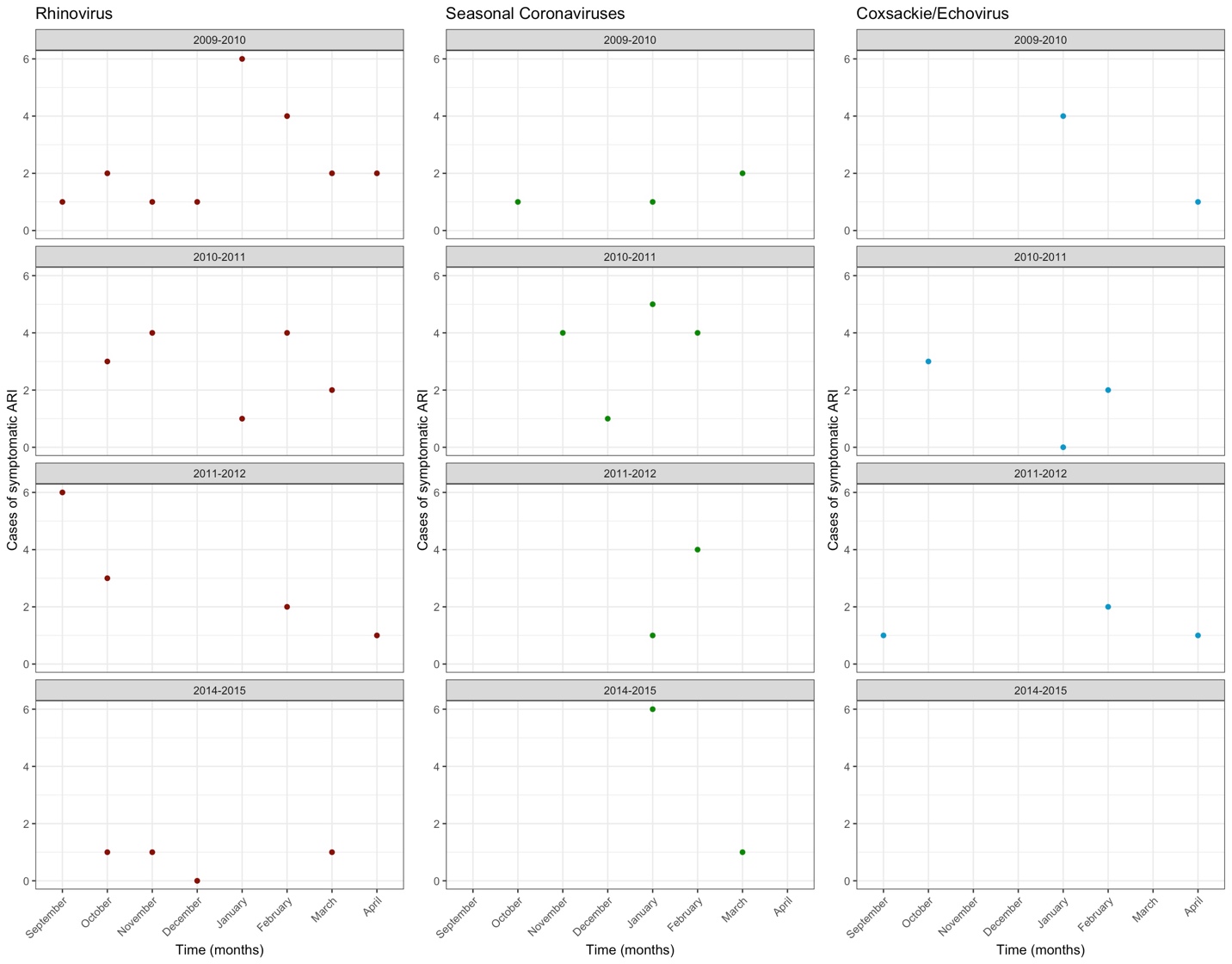
**

**APPENDICES**

**APPENDIX A. Enrollment Questionnaire**

Baseline Questionnaire - 2014 / 2015

BASE_Q0 Baseline Questionnaire

sex Please select your sex at birth:

- Male (0)
- Female (1)

race Race (check all that apply)

- American Indian or Alaskan Native (0)
- Black or African American (2)
- Native Hawaiian or Pacific Islander (3)
- Asian (1)
- White (4)
- Other (specify) (888) ____________________
- Prefer not to answer (555)

ethnicity Ethnicity:

- Hispanic / Latino (0)
- Non-Hispanic / Non-Latino (1)
- Prefer not to answer (555)

BASE_Q1 Are you currently taking any medications or drugs, prescribed or otherwise (including herbals/vitamins/supplements and over-the-counter)?

- Yes (1)
- No (0)
- Prefer Not Answer (555)

Answer If Are you currently taking any medications or drugs, prescribed or otherwise (including herbals/vit... Yes Is Selected

BASE_Q2 Please list any medications or drugs you are currently taking, prescribed or otherwise:

BASE_Q3 Do you have asthma?

- Yes (1)
- No (0)
- Don't Know / Unsure (999)
- Prefer not to answer (555)

BASE_Q4 Do you have seasonal allergies?

- Yes (1)
- No (0)
- Don't Know / Unsure (999)
- Prefer not to answer (555)

BASE_Q5 Do you currently have any other chronic medical condition(s) as diagnosed by a doctor?

- Yes (1)
- No (0)
- Don't Know / Unsure (999)
- Prefer not to answer (555)

Answer If Do you currently have any other chronic medical condition(s) as diagnosed by a doctor? Yes Is Selected

BASE_Q6 Please list your other medical condition(s) below:

1 (1)

2 (2)

3 (3)

BASE_Q7 Did you receive the flu vaccine this season (August 2013 or later)?

- Yes (1)
- No (0)
- Don't Know/Unsure (999)
- Prefer not to answer (555)

Answer If Did you receive the flu vaccine this season (August 2013 or later)? Yes Is Selected

BASE_Q8 Please select when you received the flu vaccine:

- August 2013 (1)
- September 2013 (2)
- October 2013 (3)
- November 2013 (4)
- December 2013 (5)
- January 2014 (6)
- February 2014 (7)
- March 2014 (8)
- April 2014 (9)

BASE_Q9 Have you received any other recent vaccines?

- Yes (1)
- No (0)
- Prefer not to answer (555)

Answer If Have you received any other recent vaccines? Yes Is Selected

BASE_Q10 Please list any other vaccines you have received recently:

BASE_Q11 Do you currently smoke tobacco or other products?

- Yes (1)
- No (0)
- Prefer not to answer (555)

Answer If Do you or have you ever smoked tobacco products? Yes Is Selected

BASE_Q12 How often do you smoke?

- Rarely (0)
- Weekly (1)
- Daily (2)
- Prefer not to answer (555)

BASE_Q13 Do you currently use smokeless tobacco?

- Yes (1)
- No (0)
- Prefer not to answer (555)

BASE_Q14 How often do you drink alcohol?

- Never (0)
- Rarely (1)
- Weekend (2)
- Daily (3)
- Prefer not to answer (555)

BASE_Q15 Are you currently taking or have you taken any antibiotics (e.g., amoxicillin, azithromycin/Zpac, ciprofloxacin, etc.) in the past 7 days?

- Yes (1)
- No (0)
- Prefer Not to Answer (555)

Answer If Are you currently taking or have you taken any antibiotics (e.g., amoxicillin, azithromycin/Zpac, ci... Yes Is Selected

BASE_Q16 Please list any antibiotics you have taken in the past 7 days:

1 (1)

2 (2)

3 (3)

BASE_Q17 Are you currently taking or have you taken any steroids, inhaled or oral, (ex: corticosteroid, inhaler, prednisone, albuterol, etc.) in the past 7 days?

- Yes (1)
- No (0)
- Prefer Not to Answer (555)

Answer If Are you currently taking or have you taken any steroids, inhaled or oral, (ex: corticosteroid, inhal... Yes Is Selected

BASE_Q18 Please list any steroids, inhaled or oral, that you have taken in the past 7 days:

1 (1)

2 (2)

3 (3)

**Appendix B. Daily Symptom Diary**

**
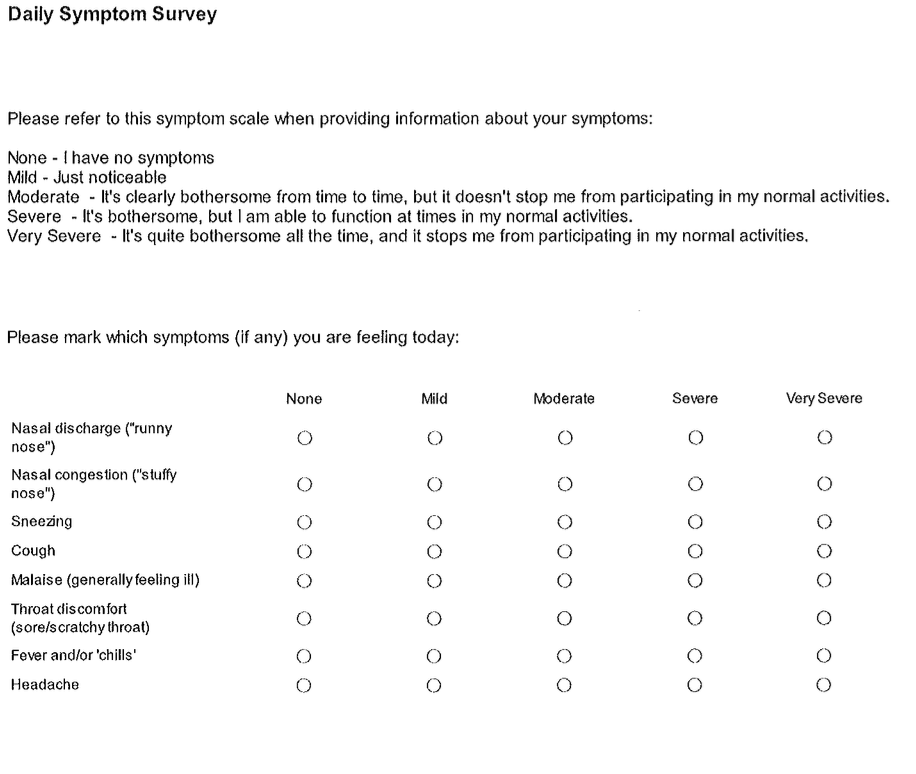
**

**
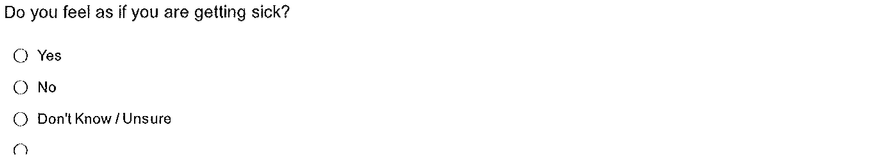
**

**
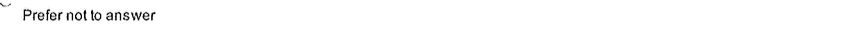
**

**
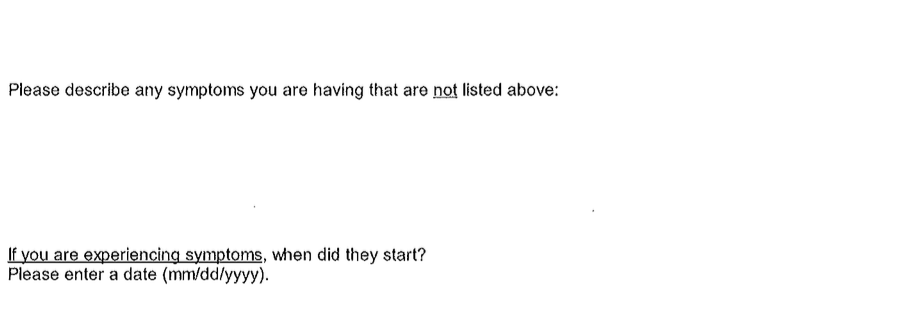
**

**
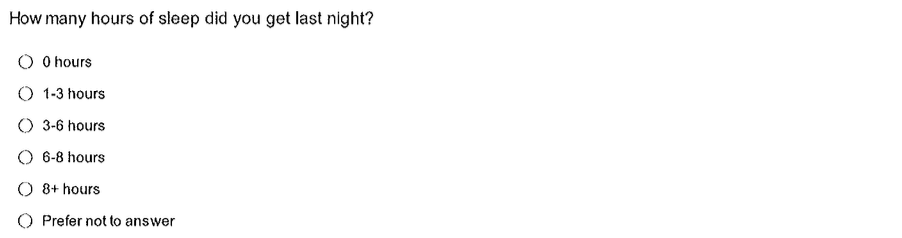
**

**Appendix C. Closeout Questionnaire**

Study Closeout Survey - 2014 / 2015

1 Did you receive the flu vaccine this season (August 2014 or later)?

- Yes (1)
- No (0)
- Don't Know / Unsure (999)
- Prefer not to answer (555)

Answer If Did you receive the flu vaccine this season (August 2013 or later)? Yes Is Selected

2 Please select the month you received your flu shot:

- August 2014 (1)
- September 2014 (2)
- October 2014 (3)
- November 2014 (4)
- December 2014 (5)
- January 2015 (6)
- February 2015 (7)
- March 2015 (8)
- April 2015 (9)

3 How many times did you get sick this year? (NOTE: "sick" is defined as any time you were ill to the point that your daily activities were impacted, such as attendance or performance at work, class, etc.)

- 0 (0)
- 1-2 (1)
- 3-4 (2)
- 5+ (3)
- Don't Know / Unsure (999)
- Prefer not to answer (555)

Answer If How many times did you get sick this year? (NOTE: "sick" is defined as any time you were ill to the point that your daily activities were impacted, such as attendance or performance at work, class,... 1-2 Is Selected Or How many times did you get sick this year? (NOTE: "sick" is defined as any time you were ill to the point that your daily activities were impacted, such as attendance or performance at work, class,... 3-4 Is Selected Or How many times did you get sick this year? (NOTE: "sick" is defined as any time you were ill to the point that your daily activities were impacted, such as attendance or performance at work, class,... 5+ Is Selected

4 Approximately how many times were your symptoms predominantly gastro-intestinal?

- 0 (0)
- 1 (1)
- 2 (2)
- 3 (3)
- 4+ (4)
- Prefer not to answer (555)

Answer If How many times did you get sick this year? (NOTE: "sick" is defined as any time you were ill to the point that your daily activities were impacted, such as attendance or performance at work, class,... 1-2 Is Selected Or How many times did you get sick this year? (NOTE: "sick" is defined as any time you were ill to the point that your daily activities were impacted, such as attendance or performance at work, class,... 3-4 Is Selected Or How many times did you get sick this year? (NOTE: "sick" is defined as any time you were ill to the point that your daily activities were impacted, such as attendance or performance at work, class,... 5+ Is Selected

5 Approximately how many times were your symptoms predominantly respiratory?

- 0 (0)
- 1 (1)
- 2 (2)
- 3 (3)
- 4+ (4)
- Prefer not to answer (555)

Answer If How many times did you get sick this year? (NOTE: "sick" is defined as any time you were ill to the point that your daily activities were impacted, such as attendance or performance at work, class,... 1-2 Is Selected Or How many times did you get sick this year? (NOTE: "sick" is defined as any time you were ill to the point that your daily activities were impacted, such as attendance or performance at work, class,... 3-4 Is Selected Or How many times did you get sick this year? (NOTE: "sick" is defined as any time you were ill to the point that your daily activities were impacted, such as attendance or performance at work, class,... 5+ Is Selected

6 Approximately how many times did your symptoms include fever?

- 0 (0)
- 1 (1)
- 2 (2)
- 3 (3)
- 4+ (4)
- Prefer not to answer (555)

Answer If How many times did you get sick this year? (NOTE: "sick" is defined as any time you were ill to the point that your daily activities were impacted, such as attendance or performance at work, class,... 1-2 Is Selected Or How many times did you get sick this year? (NOTE: "sick" is defined as any time you were ill to the point that your daily activities were impacted, such as attendance or performance at work, class,... 3-4 Is Selected Or How many times did you get sick this year? (NOTE: "sick" is defined as any time you were ill to the point that your daily activities were impacted, such as attendance or performance at work, class,... 5+ Is Selected

7 Did you become ill while residing in K-Ville?

- Yes (1)
- No (0)
- Not Applicable (2)
- Prefer not to answer (555)

8 Are you experiencing any symptoms of a respiratory infection (cold/flu) today?

- Yes (1)
- No (0)
- Prefer Not to Answer (555)

Answer If Are you experiencing any symptoms of a respiratory infection (cold/flu) today? Yes Is Selected

9 If you are experiencing symptoms, when did they start?Please enter a date (mm/dd/yyyy).

Answer If Are you experiencing any symptoms of a respiratory infection (cold/flu) today? Yes Is Selected

10 Please refer to this symptom scale when providing information about your symptoms:None - I have no symptoms Mild - Just noticeable Moderate  - It's clearly bothersome from time to time, but it doesn't stop me from participating in my normal activities Severe  - It's bothersome, but I am able to function at times in my normal activities.Very Severe  - It's quite bothersome all the time, and it stops me from participating in my normal activities.

Answer If Are you experiencing any symptoms of a respiratory infection (cold/flu) today? Yes Is Selected

11 If you are experiencing symptoms, please mark which you are feeling today:

|  | None (0) | Mild (1) | Moderate (2) | Severe (3) | Very Severe (4) |
| --- | --- | --- | --- | --- | --- |
| 1. Nasal Discharge ("Runny Nose") (1) |  |  |  |  |  |
| 2. Nasal Discharge ("Stuffy Nose") (2) |  |  |  |  |  |
| 3. Sneezing (3) |  |  |  |  |  |
| 4. Cough (4) |  |  |  |  |  |
| 5. Malaise (generally feeling ill) (5) |  |  |  |  |  |
| 6. Throat discomfort (sore/scratchy throat) (6) |  |  |  |  |  |
| 7. Fever and or 'chills' (7) |  |  |  |  |  |
| 8. Headache (8) |  |  |  |  |  |

12 Are you currently taking or have you taken any antibiotics (e.g., amoxicillin, azithromycin/Zpac, ciprofloxacin, etc.) in the past 7 days?

- Yes (1)
- No (0)
- Prefer Not to Answer (555)

Answer If Are you currently taking or have you taken any antibiotics (e.g., amoxicillin, azithromycin/Zpac, ciprofloxacin, etc.) in the past 7 days? Yes Is Selected

13 Please list any antibiotics you have taken in the past 7 days:

1 (1)

2 (2)

3 (3)

14 Are you currently taking or have you taken any steroids, inhaled or oral, (ex: corticosteroid, inhaler, prednisone, albuterol, etc.) in the past 7 days?

- Yes (1)
- No (0)
- Prefer Not to Answer (555)

Answer If Are you currently taking or have you taken any steroids, inhaled or oral, (ex: corticosteroid, inhal... Yes Is Selected

15 Please list any steroids you have taken in the past 7 days:

1 (1)

2 (2)

3 (3)

16 Have you smoked tobacco or other products in the past 7 days?

- Yes (1)
- No (0)
- Prefer Not to Answer (555)

17 The daily reminder emails were helpful.

- Disagree (1)
- Somewhat Disagree (2)
- Neutral (3)
- Somewhat Agree (4)
- Agree (5)

18 The collection times were convenient for me.

- Disagree (1)
- Somewhat Disagree (2)
- Neutral (3)
- Somewhat Agree (4)
- Agree (5)

19 The collection locations were convenient for me.

- Disagree (1)
- Somewhat Disagree (2)
- Neutral (3)
- Somewhat Agree (4)
- Agree (5)

20 Overall, my participation in the Duke Flu Study was a positive experience.

- Disagree (1)
- Somewhat Disagree (2)
- Neutral (3)
- Somewhat Agree (4)
- Agree (5)

21 Do you have any specific thoughts/opinions/comments/complaints?
