# Supplementary figures and images for: Implementation of a Prospective Index-Cluster Sampling Strategy for the Detection of Presymptomatic Viral Respiratory Infection in Undergraduate Students
Source: Open Forum Infect Dis. 2024 Feb 14;11(3):ofae081. doi: 10.1093/ofid/ofae081 (PMC10911223; doi:10.1093/ofid/ofae081)

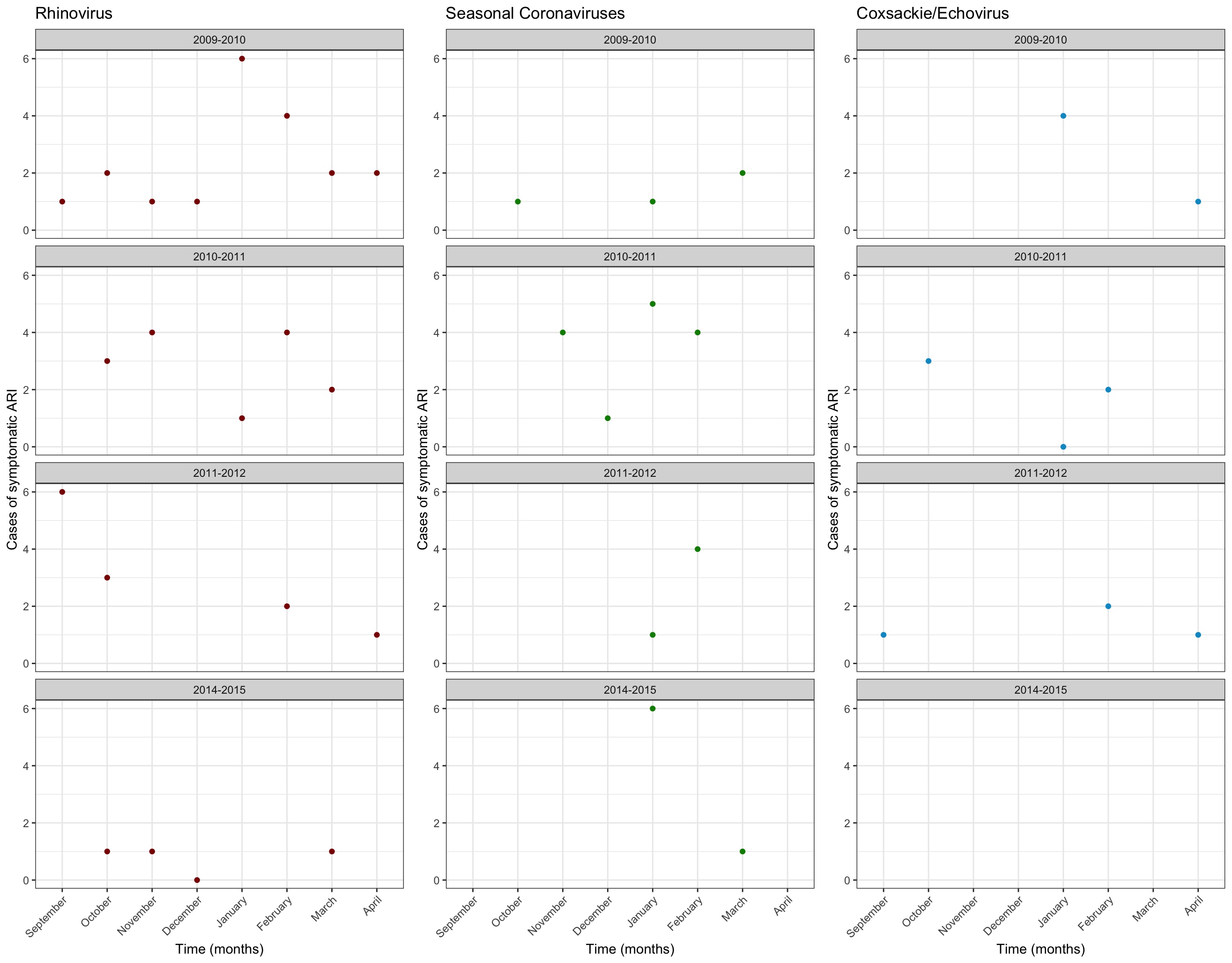

Supplement: ofae081_Supplementary_Data [file ofae081_supplementary_data.zip › Student Study_OFID_Supp Figure 4.jpeg]

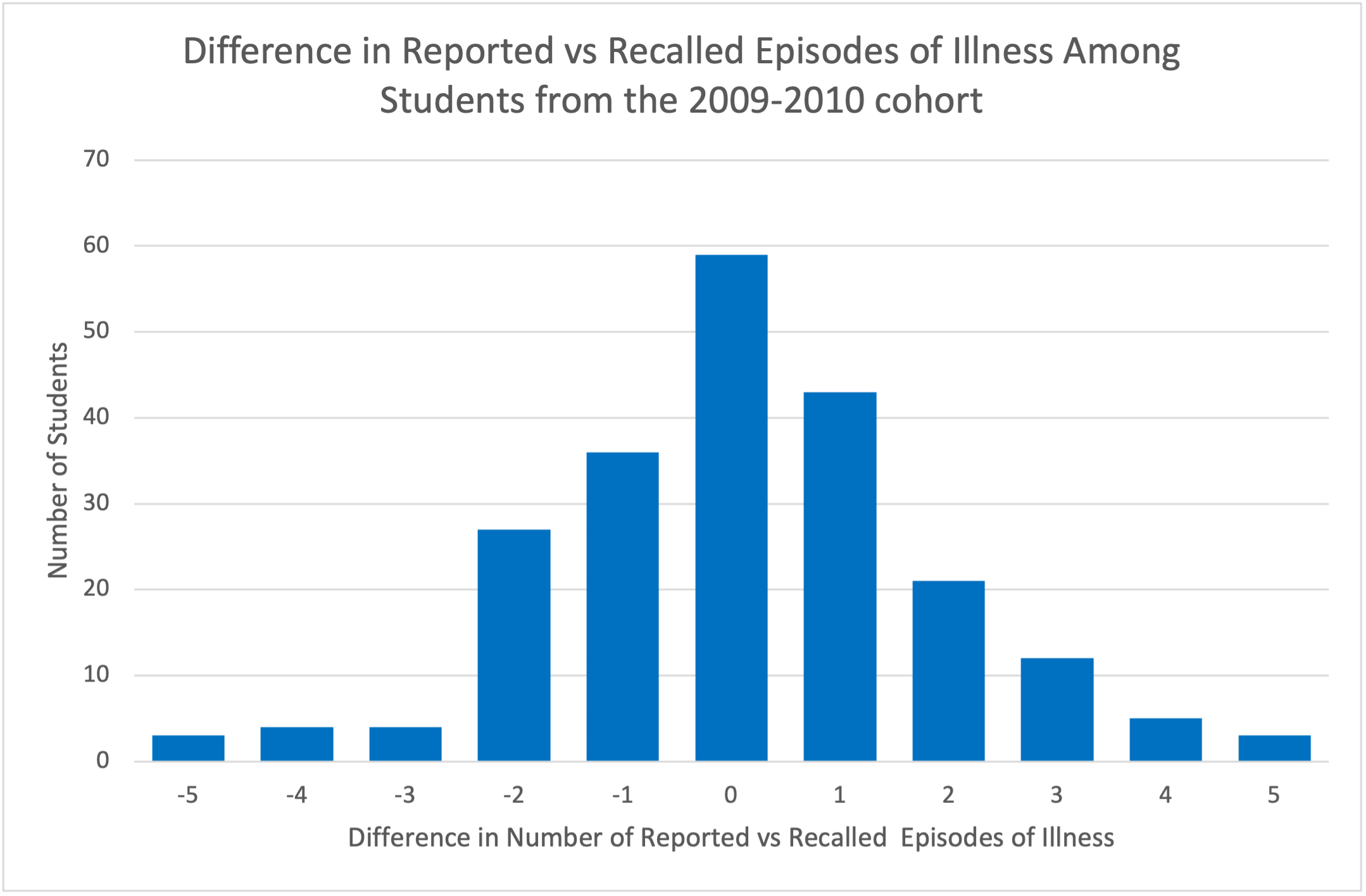

Supplement: ofae081_Supplementary_Data [file ofae081_supplementary_data.zip › Student Study_OFID_Supp Figure 1.png]
